# The disordered regions of the methyltransferase SETD2 govern its function by regulating its proteolysis and phase separation

**DOI:** 10.1016/j.jbc.2021.101075

**Published:** 2021-08-13

**Authors:** Saikat Bhattacharya, Jeffrey J. Lange, Michaella Levy, Laurence Florens, Michael P. Washburn, Jerry L. Workman

**Affiliations:** 1 Workman Lab, Stowers Institute for Medical Research, Kansas City, Missouri, USA; 2 Microscopy, Stowers Institute for Medical Research, Kansas City, Missouri, USA; 3 Proteomics, Stowers Institute for Medical Research, Kansas City, Missouri, USA; 4 Department of Cancer Biology, University of Kansas Medical Center, Kansas City, Kansas, USA

**Keywords:** histone, proteasome, chromatin, intrinsically disordered protein, protein degradation, APC, anaphase-promoting complex, CHX, cycloheximide, FDR, false discovery rate, FRAP, fluorescence recovery after photobleaching, hnRNP, heterogeneous ribonucleoprotein, IDR, intrinsically disordered region, MudPIT, multidimensional protein identification technology, ROI, region of interest, SET, Su(var)3 to 9, Enhancer-of-zeste and Trithorax, SHI, SETD2-hnRNP Interaction, SRI, Set2-Rpb1 Interaction, UPS, ubiquitin-proteasome system

## Abstract

SETD2 is an important methyltransferase that methylates crucial substrates such as histone H3, tubulin, and STAT1 and also physically interacts with transcription and splicing regulators such as Pol II and various hnRNPs. Of note, SETD2 has a functionally uncharacterized extended N-terminal region, the removal of which leads to its stabilization. How this region regulates SETD2 half-life is unclear. Here we show that SETD2 consists of multiple long disordered regions across its length that cumulatively destabilize the protein by facilitating its proteasomal degradation. SETD2 disordered regions can reduce the half-life of the yeast homolog Set2 in mammalian cells as well as in yeast, demonstrating the importance of intrinsic structural features in regulating protein half-life. In addition to the shortened half-life, by performing fluorescence recovery after photobleaching assay we found that SETD2 forms liquid droplets *in vivo*, another property associated with proteins that contain disordered regions. The phase-separation behavior of SETD2 is exacerbated upon the removal of its N-terminal segment and results in activator-independent histone H3K36 methylation. Our findings reveal that disordered region-facilitated proteolysis is an important mechanism governing SETD2 function.

The SET (Su(var)3–9, Enhancer-of-zeste and Trithorax) domain–containing enzyme, Set2/SETD2, is a crucial methyltransferase conserved from yeast to mammalian cells. It methylates lysine 36 of histone H3, a functionally important mark that suppresses cryptic transcription and is involved in DNA repair, pre-mRNA splicing, and DNA methylation (1, 2, 3, 4, 5, 6). SETD2 interacts with the large subunit of the RNA polymerase II, Rpb1, through its SRI (Set2-Rpb1 Interaction) domain, and this interaction is important for its histone methyltransferase activity (7, 8). In addition, we recently uncovered that SETD2 engages RNA-binding proteins such as the heterogeneous ribonucleoproteins (hnRNPs) through its SHI (SETD2-hnRNP Interaction) domain to couple transcription and splicing (9). Besides histone H3 methylation, SETD2 also has nonhistone substrates such as tubulin and STAT1 (10, 11). Consistent with its functionally important role, SETD2 deletion is embryonically lethal and mutations in SETD2 have been reported in many cancers, including clear cell renal cell carcinoma (12, 13, 14, 15, 16, 17, 18).

Although SETD2 deletion is detrimental to cells, maintaining its appropriate amount is also important for regulating its activity and avoiding any inadvertent consequence. We and others have shown that SETD2 protein does not accumulate in mammalian cells owing to its robust degradation by the ubiquitin–proteasome system (UPS) (19, 20). We also showed that the change in protein levels upon proteasome inhibition is not due to changes in the mRNA transcript levels (19). SETD2 has an extended N-terminal region that is absent in its yeast homolog, Set2. Also, as per the ENSEMBL database, SETD2 has a protein-coding splice variant that contains the catalytic SET domain but lacks the extended SETD2 N terminus. We previously reported that this unique N-terminal region, when removed, leads to the stabilization of SETD2, which results in a marked increase in global H3K36me3 levels in an uncharacteristic RNA Poll II–independent manner (19). Important epigenetic regulators such as DNMT3a, MutSα, and MORF depend on H3K36me3 for proper recruitment (2, 4, 21, 22). Hence, Pol II-independent H3K36me3 deposition can result in their mistargeting. In addition, methylation of tubulin by SETD2 is important for the metaphase transition (10). Furthermore, SETD2 stabilization increases cell proliferation, which is opposite to its canonical function as a tumor suppressor (19). The N-terminal segment of SETD2 regulates the requisite intracellular amount of protein, thus maintaining the fidelity of SETD2 function. How the N-terminal segment regulates SETD2 stability is unclear.

It has long been believed that the functional regions of proteins fold into a unique conformation that is crucial for a protein’s function. The polypeptide backbone of proteins also consists of amino acid sequences characterized by high net charge and low hydrophobicity. These sequences adopt extended and flexible conformations with little or no structure propensity. Such regions are known as intrinsically disordered regions (IDRs). Proteins containing localized IDRs along their whole length are highly abundant, especially in eukaryotes, and unraveling their function has been a growing subject of interest. Strikingly, pathway enrichment analysis of such proteins reveals that these IDR-containing proteins are not equally involved in all biological processes but are overrepresented in functions such as signaling and regulation (23, 24). IDRs can regulate protein function by enabling a broader spectrum of interactions, faster association kinetics, facilitating catalysis, and promoting degradation (25, 26, 27, 28).

Here we show that the SETD2 protein has IDRs that are spread across its length. Multiple such regions are targeted for degradation by the UPS, and they cumulatively decrease SETD2 half-life. We found that SETD2 can form *in vivo* liquid droplets, and this property is enhanced upon its reduced degradation. Furthermore, the stabilized SETD2 has a reduced dependency on its SRI and SHI domains for its activity. Our findings reveal that SETD2 belongs to the growing list of proteins that are regulated by their IDRs.

## Results

### Robust SETD2 proteolysis might be brought about by multiple E3 ubiquitin ligases

We previously showed that SETD2 is degraded by the UPS and the removal of its N-terminal region stabilizes the protein (19). We wanted to learn more about the UPS-mediated decay of SETD2. Previously, the E3 ubiquitin ligase SPOP and anaphase-promoting complex (APC) have been reported to promote the degradation of SETD2 protein (20, 29). Taking into consideration the robustness of SETD2 degradation, we speculated that there might be additional factors regulating SETD2 stability *in vivo*. To test this hypothesis, we first wanted to test the extent to which SETD2 is stabilized upon depleting the factors that reportedly target the protein for degradation. Measuring protein stability by tagging them with GFP has been effectively used previously to calculate protein stability index values of ∼8000 proteins (30). Hence, to check the effect of SPOP depletion on SETD2 stability, SPOP was knocked down by shRNA in GFP-SETD2 FL (full-length)-expressing 293T cells. Of the two shRNAs used, shRNA #2 treatment reduced SPOP transcript levels (Fig. S1*A*). Depletion of SPOP led to a modest increase in GFP-SETD2 FL accumulation (Fig. S1, *B* and *C*). To further substantiate this result, an SPOP Nonbinding Mutant M3 of GFP-SETD2 FL was made and expressed in WT 293T cells (Fig. S1*D*) (20). A discernible increase was not observed in the expression level of the mutant compared with the WT (Fig. S1, *E* and *H*).

Next, we looked at the possible role of APC in regulating SETD2 degradation. The APC recognizes the D and KEN Box in a protein and targets them for degradation by the UPS (25). SETD2 has a putative D Box at position 2033 to 2035 and KEN Box at 2078 to 2081 (Fig. S1*F*). Both these regions were mutated in GFP-SETD2 FL and the SETD2 fragment GFP-SETD2 N4 (1964–2564), where the D and KEN boxes are located. The expression of these mutants was tested in 293T cells (Fig. S1, *E*, *G*, and *I*). Comparison of the mutant with the WT expression did not reveal any discernible difference. Besides, both the GFP-SETD2 FL SPOP Nonbinding Mutant M3 and the D-KEN box mutants retained sensitivity to MG132 (Fig. S1, *E* and *G*–*I*).

In conclusion, our results agreed with the previously published data that SPOP partially regulates SETD2 stability and also indicated that the posttranslational control of SETD2 abundance is more complicated than previously thought and may contain several redundancies.

### SETD2 has multiple long intrinsically disordered regions

The NCBI Conserved Domain Search (31) revealed that the SETD2 N terminus that destabilizes the protein lacks any known domains or motifs and did not yield any clue about its possible function. Also, the SETD2 N terminus has very little sequence similarity to other known protein sequences. Consequently, homology modeling of the structure using SWISS-MODEL (32) failed. Furthermore, the sequence is 81% disordered based on Robetta prediction (http://robetta.bakerlab.org/) (33).

**Figure 1:**
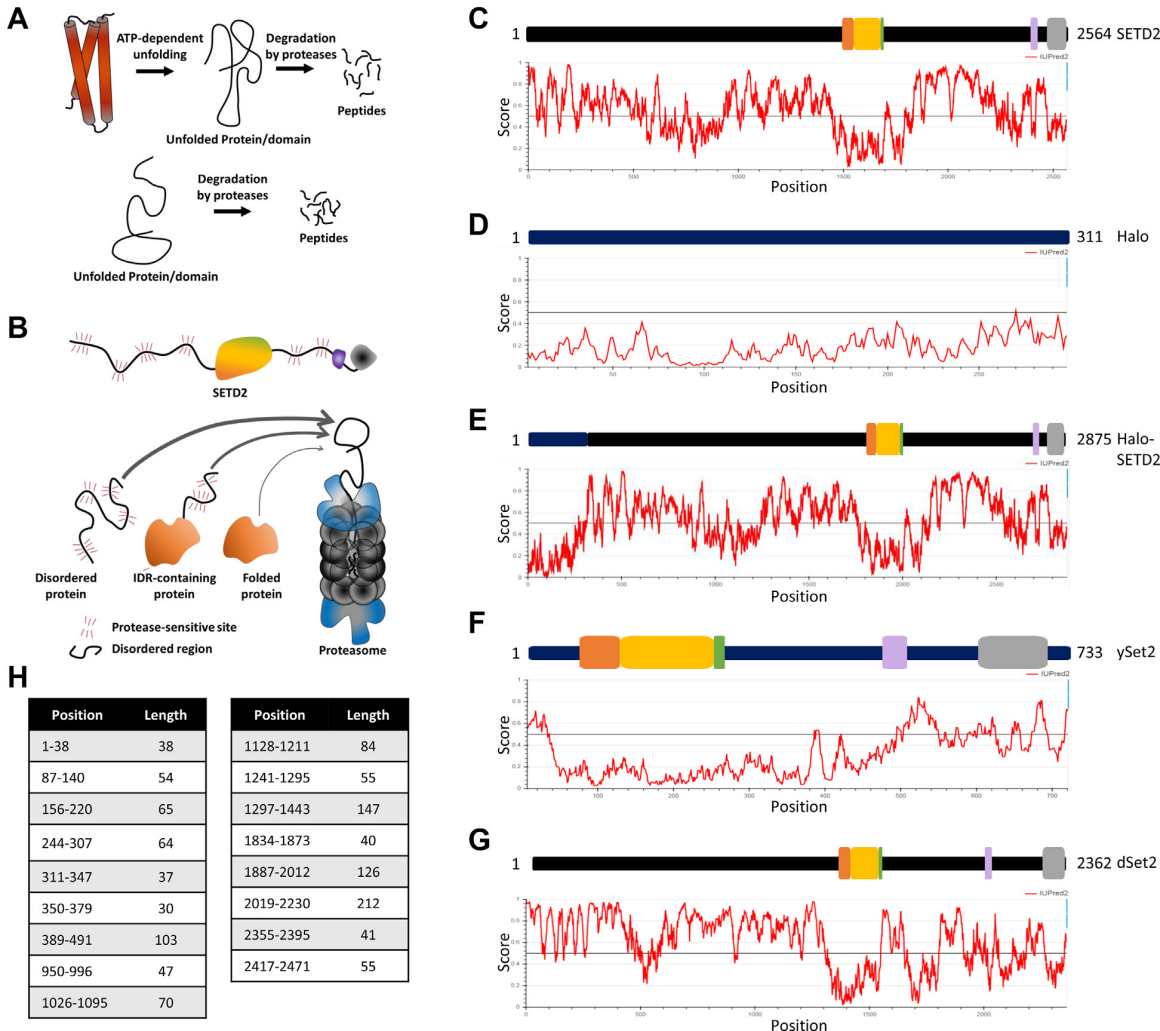
**SETD2 is rich in intrinically disordered regions.***A*, illustration depicting that unfolded proteins and domains can directly be degraded by proteases without requiring ATP-dependent unfolding**.***B*, *cartoon* illustrating that disordered proteins can gain access to the catalytic core of the proteasome more readily and hence can be degraded more robustly. The thickness of the *arrow* shows the ease of access to the proteasome. The *red whiskers* depict the sensitivity to proteolysis. *C*–*G*, SETD2 has intrinsically disordered regions. Various protein sequences were subjected to disordered region prediction using IUPRED2A. See the text for more details. *H*, position and length of disordered regions >30 amino acids in SETD2.

There is emerging evidence to support a role for structural features of substrates such as the IDRs in influencing their half-life (34, 35). In fact, susceptibility to proteolysis is one of the features commonly associated with proteins with IDRs possibly because they are easy targets for proteases (36, 37). Substrate denaturation enhances the rates of proteolysis (38). Folded proteins require denaturation prior to efficient proteolytic degradation, whereas IDRs are inherently sensitive to proteolysis without the need for prior denaturation (Fig. 1*A*). Consistent with this, proteolytic cleavage sites tend to overlap with regions for which electron density in crystal structures is absent, suggestive of inherent disorder (39). Access to the proteasome is an important step that regulates the half-life of protein substrates (40, 41). The catalytic residues are situated deep within the proteasome core particle and are only accessible through a long narrow channel. It is easier for disordered segments to gain access to the proteasome as compared with folded regions of a protein (Fig. 1*B*). Hence, we decided to perform a deeper analysis of the disordered regions in the SETD2 protein.

Prediction of the disordered region in SETD2 was performed using IUPRED2 (42). IUPred provides a score between 0 and 1 for every residue, with a score greater than 0.5 meaning the residue is more likely part of a disordered region. Strikingly, 1546 of the 2564 (60.29%) residues of the SETD2 protein returned a score of >0.5 (Fig. 1, *C* and *E*). In comparison, almost all the residues of a well-folded Halo protein that was used as control returned a score of <0.5 (Fig. 1, *D* and *E*). Furthermore, 67.6% of residues in the N terminus scored >0.5 as compared with 51.4% of residues in the C terminus.

Besides the overall level of disorder, long N-terminal and internal disordered segments contribute to short protein half-life *in vivo* (34, 43). A critical minimum length of ∼30 residues allows a disordered terminus of a ubiquitinated substrate to efficiently initiate proteasomal degradation (34, 44, 45). Hence, we next looked for long disordered regions (>30 residues) in SETD2. Seventeen such stretches were found in SETD2 of which 11 were in the N-terminal region, 5 in the C-terminal fragment, and 1 overlapping the junction between the N- and the C-terminal segments (Fig. 1*H*).

Our findings regarding the stability and disordered region prediction of SETD2 suggest the possibility that the IDRs of SETD2 may govern its half-life.

### Multiple disordered segments of SETD2 have a combined effect on its half-life

**Figure 2:**
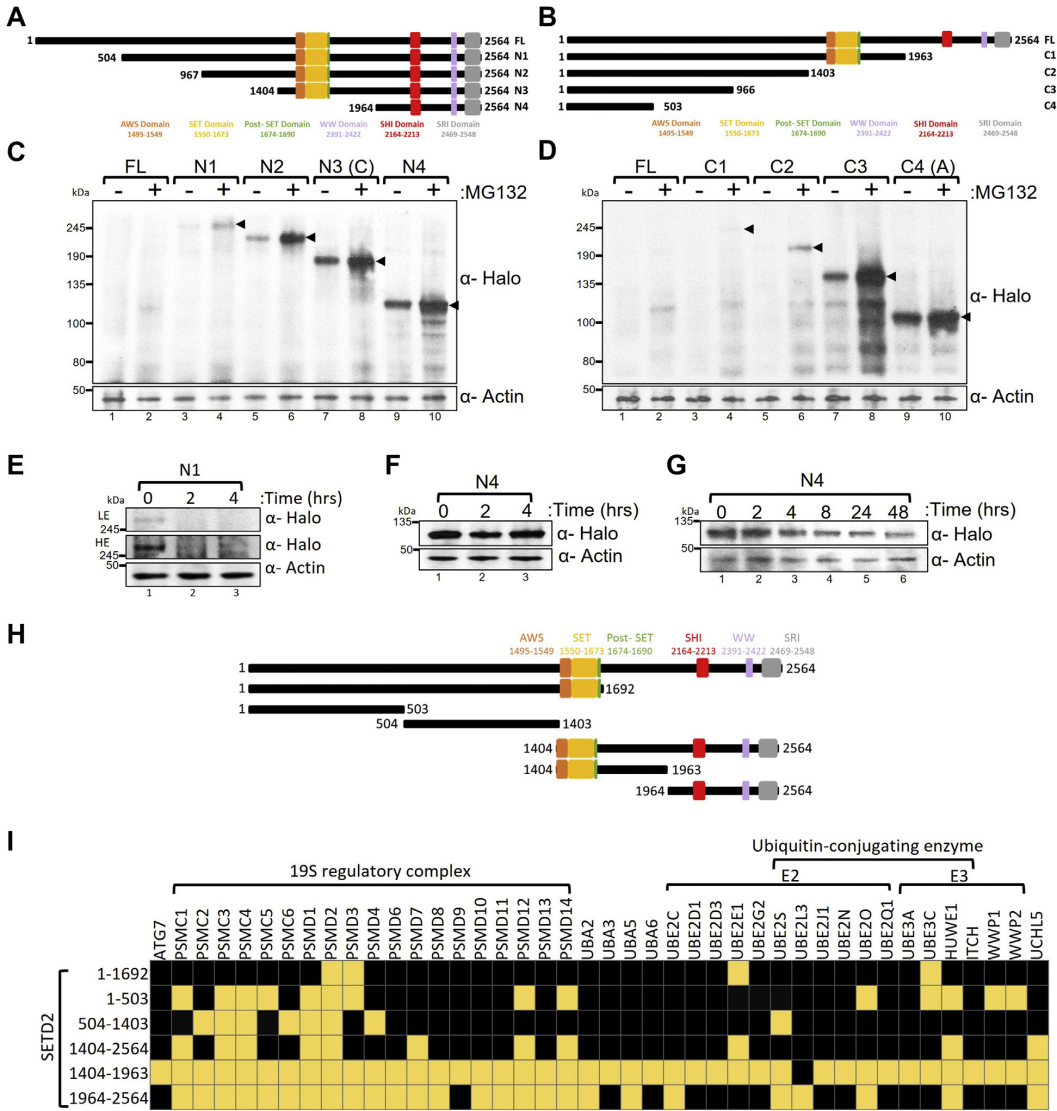
**Deleting SETD2 fragments leads to its stabilization.***A* and *B*, *cartoon* illustrating the N- and C-terminal truncations of SETD2 along with its known domains. *C* and *D*, western blot of whole-cell lysates probed with the depicted antibodies. Lysates of 293T cells expressing Halo-SETD2 constructs were prepared after 12 h of MG132 (10 μM) treatment. The expected bands for the target protein are indicated by *arrows*. *E*–*G*, western blot of whole-cell lysates probed with the indicated antibodies. Lysates of 293T cells expressing Halo-SETD2 constructs were prepared after cycloheximide (10 μM) treatment. The duration of the treatment is shown in the figure. *H*, *cartoon* illustrating the full-length SETD2 and its truncations that were used for affinity purification from 293T cells. *I*, heatmap showing the components of the UPS that were affinity purified with SETD2. A *yellow box* indicates that the UPS component was identified in mass spectrometry analysis, whereas the *black box* represents the UPS component was not detected. HE, high exposure; LE, low exposure; UPS, ubiquitin–proteasome system.

Proteins with several internal disordered segments have shorter half-lives than proteins with only one such segment (34). SETD2 undergoes robust degradation and is predicted to have numerous disordered segments throughout (Fig. 1). If the multiple disordered segments of SETD2 collectively enhance its proteolysis, we predicted that the protein levels of the shorter fragments will be higher than that of the long ones. To test this, a series of constructs were made to express Halo-tagged N- or C-terminal truncations of SETD2 in 293T cells (Fig. 2, *A* and *B*). The use of recombinant truncation mutants to determine the region of protease sensitivity in a protein is a commonly used approach and has been successfully used previously (20, 46). Western blotting of whole-cell extracts with an anti-Halo antibody revealed that, for both the N- and C-terminal truncations, SETD2 protein levels anticorrelated with its length (Fig. 2, *C* and *D*). In fact, the difference in the expression level of SETD2 FL *versus* N4 and C4 was so marked that, at the exposure level in which the signal for bands corresponding to N4 and C4 were saturated, FL was not detectable even upon MG132 treatment (Fig. 2, *C* and *D*). This is in agreement with our previous report where we showed that three nonoverlapping fragments of Halo-SETD2, N1a (C4), N1b (504–1403), and C (N3), expressed robustly in 293T cells, indicating that smaller fragments of SETD2 accumulate to higher levels than SETD2 FL and is consistent with our hypothesis (19).

To test whether strong overexpression induced by the CMV promoter contributes toward the expression pattern observed, we used a truncated CMVD2 promoter that shows a much weaker activity as compared with the full-length CMV promoter. Previously we have used this approach to regulate the expression of SETD2 protein (19). Consistent with the data that despite using the strong CMV promoter the expression of FL and N1 were barely detectable (Fig. 2*C*), expression of these constructs could not be detected using the truncated CMVD2 promoter (Fig. S2*A*). The expression of the smaller SETD2 fragments C4 and N4 was observed, demonstrating that even upon significantly reduced expression conditions, the accumulation of smaller SETD2 fragments is markedly higher than that of the larger ones (Fig. S2*A*). Furthermore, to rule out the possible effect of a large epitope like Halo or GFP on SETD2 expression, we tested the expression of a few of the SETD2 constructs using FLAG tag and scored the expression level using anti-FLAG antibody in Western blotting. The data recapitulated our observation using the Halo or GFP-tagged version of the proteins. The smaller SETD2 fragments N4 and 967 to 1403 accumulated to a much greater extent than the larger N3 fragment, whereas the expression of FL was not detectable at that exposure level (Fig. S2*B*). Furthermore, to confirm that the differences observed in the SETD2 levels are indeed due to the shorter half-life of larger SETD2 fragments, a time-chase experiment was performed to monitor the levels of Halo-N1 and N4 upon the treatment of cells with cycloheximide (CHX), a protein translation inhibitor. Post 2 h CHX treatment, the larger Halo-N1 could no longer be detected in the whole-cell lysates, whereas no appreciable decrease was observed for the smaller fragment Halo-N4 even after 4 h (Fig. 2, *E* and *F*). Of note, Halo-N4 could be readily detected even after 48 h of CHX treatment (Fig. 2*G*), demonstrating that the shorter fragment N4 has a longer half-life in comparison with N1.

These experiments demonstrate that SETD2 does not have a single hypersensitive region for proteolysis. Rather its different segments co-operatively regulate its half-life.

### SETD2 IDRs have a direct effect on its half-life

We wanted to investigate whether, besides IDRs, other intrinsic parameters of the SETD2 sequence may also contribute to its degradation. Short peptide motifs such as PEST sequences may also lead to robust protein degradation (47). Sequence analysis of SETD2 revealed the presence of multiple PEST motifs across its sequence with a score of more than five, which is considered significant (Fig. S3, *A* and *B*). The highest score of 26.89 was found in the stretch of 504 to 1403 residues (Fig. S3*A*). To test the possible contribution of PEST motifs in governing SETD2 degradation, we used a GFP-tagged construct of SETD2 504 to 1403 with an NLS (GFP-504-1403′), which we have previously used (19). The PEST motif from GFP-504-1403′ was deleted, and its expression was checked in 293T cells by fluorescence microscopy and Western blotting of whole-cell extracts with anti-SETD2 antibody (Fig. S3, *C*–*E*). The expression of the segment GFP-504-1403′ with and without the PEST motif was similar. This is consistent with a study that found that disordered segments have direct effects on half-life by forming initiation sites for degradation by the proteasome rather than acting indirectly by embedded destruction signals (34, 48, 49, 50).

The expression data of SETD2 truncation mutants revealed that all the SETD2 fragments display sensitivity to proteasome inhibition by MG132 treatment, suggesting that all the segments contribute to SETD2’s UPS-mediated decay (Fig. 2, *C* and *D*). To further support this observation, we wanted to test whether the SETD2 segments can engage with UPS components independent of one another. For this, Halo-tagged SETD2 truncation mutants were affinity purified, and the purified protein complexes were subjected to multidimensional protein identification technology (MudPIT) mass spectrometry to identify UPS components (Fig. 2*H* and Table S1). MudPIT revealed that numerous subunits of the 19S regulatory complex of the proteasome as well as E2 and E3 ubiquitin ligase were copurified with SETD2 fragments (Fig. 2*I*, yellow boxes). Previously, we and others have shown that full-length SETD2 is difficult to purify because of its short half-life and this challenge can be overcome by purifying its smaller fragments as they accumulate more easily (9, 51). That the larger number of UPS components copurified with the smaller fragments 1 to 504, 505 to 1403, 1404 to 1963 and,1964 to 2564 as compared with 1 to 1692 and 1404 to 2564 is likely due to the greater feasibility of purifying smaller SETD2 fragments resulting from their higher stability.

To conclude, numerous regions of SETD2 were found to co-operatively regulate its UPS-mediated proteolysis and this is consistent with the known combined effect of multiple IDRs on reducing protein half-life.

### The IDR-rich N terminus of SETD2 can reduce the half-life of ySet2

**Figure 3:**
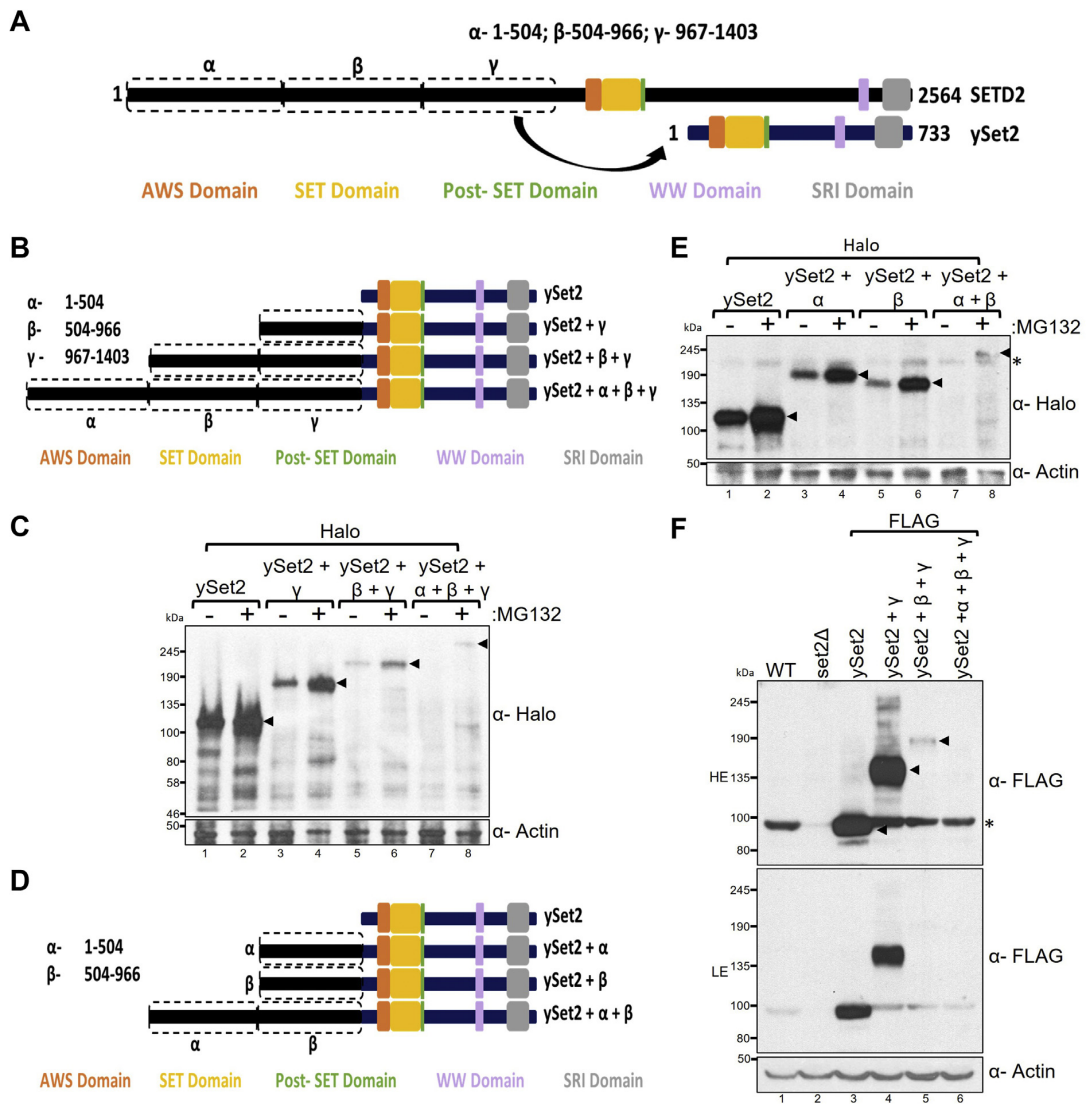
**The SETD2 N terminus can destabilize ySet2.***A*, *B* and *D*, *cartoon* illustrating the chimeric ySet2 constructs made by fusing with the segments of N terminus of SETD2. *C* and *E*, western blot of whole-cell lysates probed with the depicted antibodies. Lysates of 293T cells expressing Halo-ySet2 constructs were prepared after 12 h of MG132 (10 μM) treatment. The expected band for the target protein is depicted by *arrows*. *F*, western blot of whole-cell lysates of yeast BY4741 expressing FLAG-ySet2 constructs (using pRS416) probed with the antibodies indicated. ∗, nonspecific; HE, high exposure; LE, low exposure.

We previously showed that, when yeast Set2, ySet2, was expressed in mammalian cells, it localized to the nucleus and its levels were significantly higher than SETD2 FL and are comparable with SETD2 C (N3) with which it shares homology (19). Disordered region prediction revealed that overall ySet2 is a well-ordered protein with a much lower proportion of residues (24.69%) predicted to be disordered as compared with its homolog SETD2 (Fig. 1, *C* and *F*). We speculated that the disparity in expression between ySet2 and SETD2 could be due to the differences in the disordered region abundance between the two proteins. Hence, we wanted to test whether the N-terminal disordered segment of SETD2 can destabilize the ySet2 protein when fused to it (Fig. 3*A*).

Thus, constructs were made to express Halo-tagged chimeric proteins that have increasing stretches of the N-terminal segment of SETD2 (α: 1–504, β: 504–966, and γ: 967–1403) fused upstream of ySet2 (Fig. 3*B*). The expression of these constructs was tested in 293T cells by probing whole-cell lysates with an anti-Halo antibody. A progressive decrease in the accumulation of chimeric ySet2 was observed with the increasing length of the N-terminal segment, and all the chimeras displayed sensitivity to MG132 treatment (Fig. 3*C*). Next, we wanted to test whether the destabilization brought about by the SETD2 N terminus is influenced by the order in which the fragments are added upstream of ySet2. For this, the expression of a different set of chimeric constructs was tested in 293T cells (Fig. 3, *D* and *E*). The addition of α and β destabilized ySet2 and fusing α + β destabilized it further, even in the absence of fragment γ. The data also suggest that the SETD2 fragments co-operatively regulate stability, which is consistent with the findings that IDRs have a combined effect on protein half-life.

### The effect of SETD2 N terminus on protein half-life in yeast suggests evolutionary conservation of IDR-mediated degradation

ySet2 half-life is regulated by the proteasome (52). The architecture of the proteasome is conserved from yeast to mammals (44, 45). Consistent with that, we previously showed that ySet2 responds to MG132 treatment in 293T cells, suggesting that it is targeted for degradation through UPS in human cells as well (19). We wondered whether the IDR-rich SETD2 N-terminal segment from humans can enhance ySet2 proteolysis in yeast. To test this, the expression of FLAG-tagged WT and chimeric ySet2 proteins was checked in the yeast strain BY4741. Protein levels of ySet2 were scored by probing the whole-cell extracts with an anti-FLAG antibody. Although the addition of the SETD2 fragment γ (967–1403) did not have an impact, the addition of β and α + β had a drastic destabilization effect on the ySet2 protein in yeast cells (Fig. 3*F*).

Collectively, our results demonstrate that the IDR-rich N terminus of the SETD2 protein has a destabilization effect on both SETD2 and its yeast homolog ySet2. They also suggest evolutionary conservation of the role of the N-terminal region of SETD2 in bringing about the degradation of a protein without the need for specific E3 ubiquitin ligases. This is key evidence for IDR-mediated degradation where IDRs directly act as efficient sites for initiating proteasomal degradation (34).

### SETD2 with reduced IDRs exhibits unexpected methyltransferase activity

At high cellular levels, SETD2 has a reduced dependency on SRI domain–mediated activation for H3K36me3 deposition (19). This possibly is one reason why cells require a robust mechanism for SETD2 turnover so that its spurious methyltransferase activity is kept in check. We recently showed that, in addition to the SRI domain, the SHI domain is also important for the activation of SETD2 (9). Deletion of the SRI and SHI domains leads to a global decrease in H3K36me3 levels and a SETD2 mutant lacking both of these domains has almost no activity (9). We wanted to test whether the IDR-mediated degradation of SETD2 is required to maintain its dependency on SHI domain–mediated activation as well.

**Figure 4:**
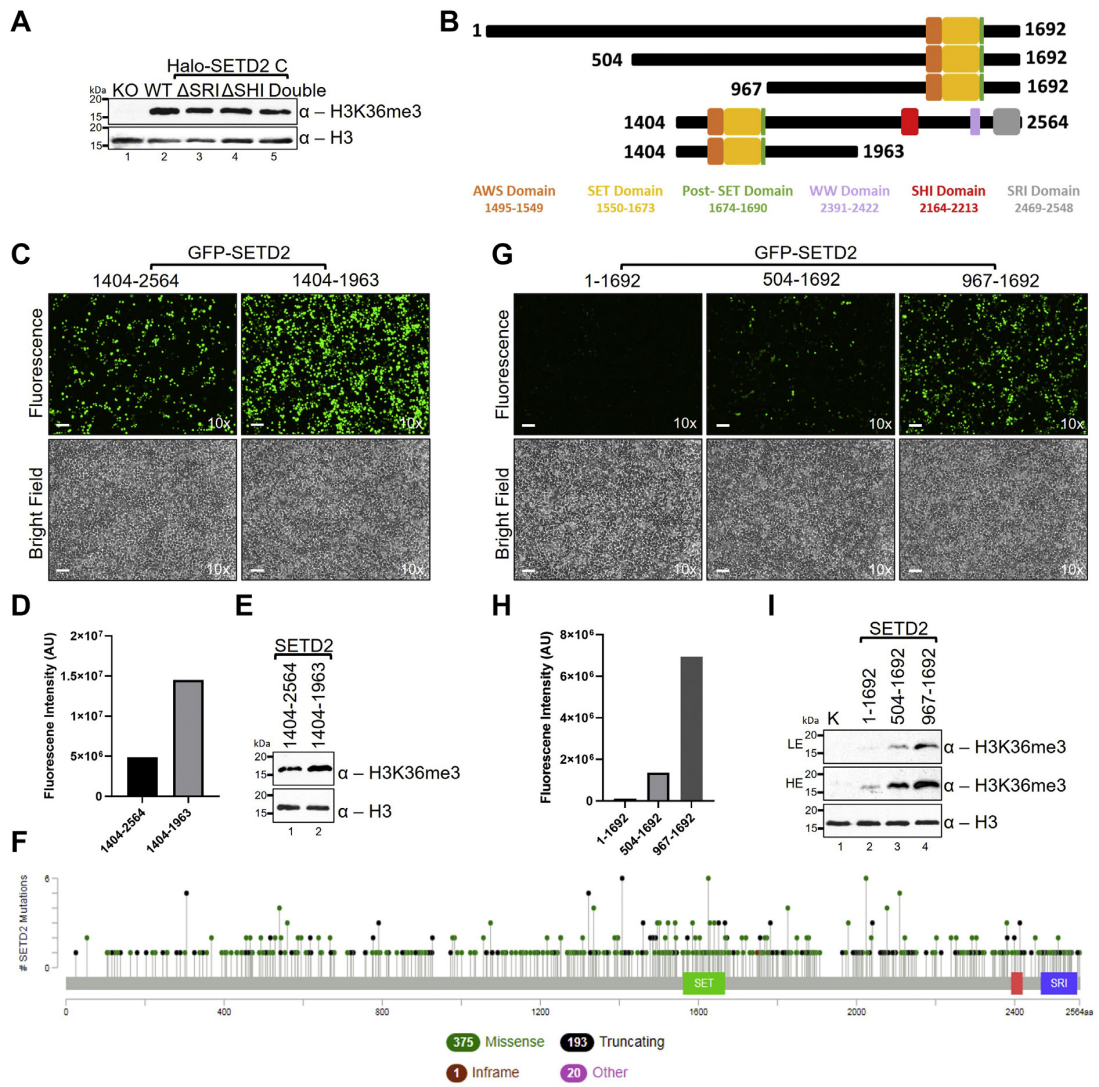
**Stabilized SETD2 loses dependency on it activators for H3K36me3 deposition.***A*, western blotting of whole-cell extracts with the indicated antibodies. *B*, *cartoon* showing SETD2 truncations that were used in the experiments for the panels below. *C*, *D*, *G* and *H*, microscopy images and their quantification of cells expressing GFP-SETD2 constructs, and *E* and *I*, western blotting of whole-cell extracts with the indicated antibodies. The scale bar represents 1 mm. *F*, *cartoon* from http://www.cbioportal.org depicting the location of various mutations found in the SETD2 gene of patients with cancer that may result in the generation of truncated forms of SETD2 with altered half-lives.

To test this, Halo-SETD2 C mutant constructs lacking the N-terminal disordered region (1–1403) as well as the SRI and SHI domains were introduced in *setd2Δ* 293T (KO) cells in which the exon 3 of SETD2 has been disrupted (53). Next, whole-cell extracts were prepared and H3K36me3 levels were analyzed by Western blotting. Consistent with the fact that SETD2 is the sole enzyme that is responsible for H3K36me3 modification in mammalian cells, KO cells did not have this mark, whereas KO cells that were rescued with Halo-SETD2C had robust H3K36me3 signal (Fig. 4*A*). The ΔSRI, ΔSHI, and Double (ΔSRI + ΔSHI) mutants also exhibited robust H3K36me3 activity, suggesting that at a high expression level SETD2 has reduced dependency on its activators for modifying histones (Fig. 4*A*).

Of interest, in the ENSEMBL database, SETD2 has nine splice variants of which three are protein coding. One of those with transcript ID ENST00000638947.1 codes for a 591-residue segment of SETD2 (1451–2039) that, importantly, contains the catalytic SET domain. This segment lacks the IDRs present in full-length SETD2 and is expected to robustly accumulate. To test this possibility, we introduced GFP-SETD2 1404 to 1963 in KO cells and analyzed H3K36me3 level by Western blotting (Fig. 4*B*). Consistent with our findings so far, the shorter 1404 to 1963 fragment expressed strongly, even higher than SETD2C (1404–2564), and demonstrated robust H3K36me3 activity (Fig. 4, *C*–*E*).

Besides naturally occurring splice variants, many missense and truncation mutations that can potentially alter the half-life are found in SETD2 of patients with cancer (Fig. 4*F*). Many such mutations can potentially result in the expression of SETD2 truncations, including ones that have the N-terminal region and catalytic domains intact but are missing the rest of SETD2’s activation domains (SRI and SHI). To test whether such mutants also might have unexpected H3K36me3 activity, we tested the expression of one such possible mutant (SETD2 1–1692) and its smaller truncations in KO cells (Fig. 4*B*). Indeed, the expression of these fragments reiterated our observations that SETD2 progressively becomes more stable on becoming shorter with an accompanying increase in its ability to deposit H3K36me3 with reduced dependency on its activators (Fig. 4, *G*–*I*).

### SETD2 puncta are dynamic phase-separated bodies

**Figure 5:**
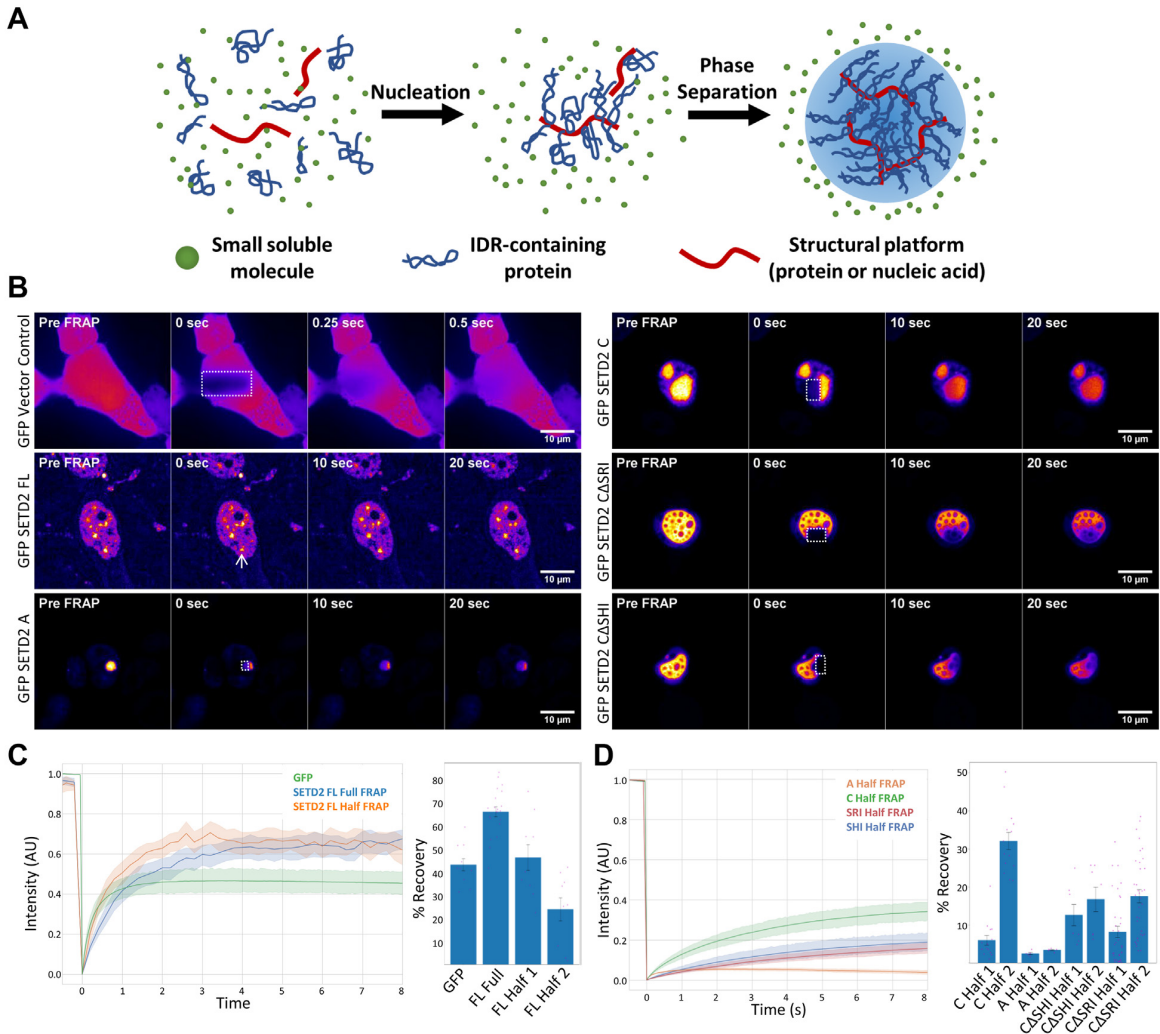
**SETD2 phase separates *in vivo*.***A*, illustration showing phase separation of IDR-containing proteins. *B*, microscopy images showing different time points of the FRAP experiments. The scale bar represents 10 μm. *C* and *D*, plots and bar graphs showing the recovery of fluorescence in the bleached region of interest.

We previously reported that GFP-SETD2 FL as well as the endogenous SETD2 form puncta that are reminiscent of *in vivo* liquid droplets and are not nucleolar (19). Besides contributing to robust protein degradation, IDRs serve as flexible platforms for protein–protein interactions and are the driving force behind biological liquid–liquid phase transitions (54, 55, 56, 57, 58). IDR-containing proteins interact with a structural platform such as protein or RNA, which leads to the nucleation event (Fig. 5*A*). Further interactions of nonstructured domains with each other may result in the formation of phase-separated liquid droplets (Fig. 5*A*) (58).

As IDRs are present across the length of SETD2, we were curious which region is responsible for its ability to form *in vivo* condensates. Microscopy visualization of 293T cells expressing GFP-SETD2 truncations (described in Fig. 2) revealed that all SETD2 fragments formed puncta to some extent, especially upon inhibition of its proteolysis (Fig. S4*A*: In this case, exposure settings were unique to each panel, as the purpose is to show the puncta clearly. For quantitative purposes, Western blots [Fig. 2] were performed).

To test whether the aggregates formed by SETD2 are dynamic structures, we performed half florescence recovery after photobleaching (FRAP) of puncta formed by SETD2. In half FRAP, half of the puncta is bleached and the fluorescence recovery in the region of interest (ROI) is monitored. Half FRAP is a widely used assay to determine the molecular dynamics and mobility of *in vivo* droplets (59). For liquid-like droplets, the fluorescence recovery is quick. The fluorescence recovery is slow in gel-like droplets, and there is no recovery in fluorescence signal in solid protein aggregates after photobleaching (60). An additional component of the fluorescence recovery is the addition of nucleoplasmic SETD2 protein into the SETD2 puncta. To separate the contribution of internal recovery and the addition of new protein, we also performed full FRAP. For full FRAP, the curves were well fit by a single exponential. This yielded the tau value, which represents the diffusion time, of the addition of new protein from the nucleoplasm into the puncta. For half FRAP, the curves were well fit by a double exponential. The slow component of this fit was fixed to the full FRAP tau value for each sample. In this case, the fast component of the recovery fits the internal turnover inside the puncta, whereas the slow component fits the addition of protein from the nucleoplasm to the puncta.

In both half and full FRAP experiments, GFP-SETD2 full-length protein recovers very quickly on a time scale that is almost as fast as cytoplasmic GFP (Fig. 5, *B* and *C* and Fig. S5, *A* and *C*). This indicates that there is robust internal diffusion of SETD2 protein as well as the addition of nucleoplasmic SETD2 to the puncta. Both these behaviors are consistent with these puncta being true phase-separated liquid droplets.

We next wanted to investigate which region of SETD2 is responsible for its phase separation. Previously we found that GFP-SETD2 A (fragment C4 in Fig. 2) and SETD2 C (fragment N3 in Fig. 2) fragments were the most aggregate prone (19). Colocalization experiments of RFP-Ub with GFP-SETD2 revealed that the N-terminal SETD2 A fragment shows a strong colocalization with ubiquitin, but the C-terminal region does not, suggesting that the puncta formed by the two fragments might have different properties (Fig. S4*B*). To investigate this further we performed half and full FRAP on these fragments. GFP-SETD2 A puncta showed little fluorescence recovery in full and half FRAP experiments indicating a solid-like material with little addition of new protein (Fig. 5, *B* and *D* and Fig. S5, *B*–*F*). Strikingly, GFP-SETD2 C puncta recovered fluorescence robustly in both full and half FRAP, which indicates that these puncta are liquid-like (Fig. 5, *B* and *D* and Fig. S5, *B*–*F*). The rate at which GFP-SETD2 C puncta recovered in both full and half FRAP was slower than the full-length protein (Fig. S5, *A*–*F*). This indicates that these aggregates are more viscous and that the addition of new protein to the aggregate was slower than the puncta formed by SETD2 FL. In addition, there was a marked difference in size between the puncta formed by SETD2 FL *versus* C.

These experiments demonstrate that the C-terminal segment of SETD2 can form liquid droplets, whereas its N-terminal segment forms solid aggregates (see Discussion). Hence, the removal of the N-terminal region leads to the formation of larger *in vivo* liquid droplets by SETD2.

### Pol II and hnRNP interactions are not required for SETD2 phase separation

We found that the C-terminal segment of SETD2 that harbors its characterized domains can form liquid droplets, but its N-terminal segment does not. The cytoplasmic mutant of SETD2 C does not form puncta, suggesting that SETD2’s intrinsic sequence characteristics are not sufficient for its phase separation and additional factors are required (Fig. S5*G*). The C-terminal segment of SETD2 contains its known functional domains including the catalytic domain as well as the SRI and SHI domains that mediate its interaction with RNA Pol II and the hnRNPs, respectively. To test whether interaction with these proteins is required for SETD2’s phase separation, we performed FRAP of SETD2 mutants lacking the SRI (SETD2CΔSRI) and the SHI (SETD2CΔSHI) domains. Both the GFP-SETD2 CΔSHI and GFP-SETD2 CΔSRI samples showed fluorescence recovery in both full and half FRAP with intermediate rates of recovery that also show viscous liquid-like aggregates with slow protein addition from the nucleoplasm (Fig. 5, *B* and *D* and Fig. S5, *B*–*F*). The GFP-SETD2 CΔSRI samples showed the most heterogeneity in puncta morphology and full/half FRAP recovery. The reason for this heterogeneity is beyond the scope of this study. However, the fact that the CΔSRI and CΔSHI mutants are still liquid-like and form *in vivo* liquid droplets indicates that factors besides interaction with RNA Pol II and hnRNPs contribute to SETD2’s phase separation.

## Discussion

Although we and others have reported the role of the proteasome in regulating SETD2 stability, the determinants of such robust SETD2 degradation are not clear. We not only show that the numerous IDRs of SETD2 are targeted for proteasomal degradation but for the first time also reveal that SETD2 can form liquid droplets *in vivo*.

### SETD2 IDRs potentially act as efficient sites for initiating proteasomal degradation

The correct turnover of proteins is important for maintaining cellular homeostasis and function. Protein substrates are targeted for degradation through polyubiquitination by ubiquitin ligases (61, 62). Also, intrinsic structural features of substrates such as IDRs are known to regulate the half-life of proteins such as ODC, Rpn4, TS, p53, p21, c-Jun, and α-synuclein (63, 64, 65, 66). Selective degradation of proteins plays an important regulatory role in dynamic processes such as signal transduction, transcription, and cell cycle control. In fact, an analysis of the functional annotations of proteins with long disordered segments revealed enrichment for associations with regulatory and transcription functions (34). This suggests that the IDR-mediated regulation of protein half-life that we discovered for SETD2 might be a prevalent mechanism employed by cells to govern essential processes.

Autophagy and degradation by the UPS are the two major mechanisms for protein turnover in eukaryotic cells (67). However, little is known about IDR-mediated degradation by autophagy. Previously, we showed that the inhibition of autophagy by treatment of cells with the lysosome inhibitor chloroquine did not have any effect on SETD2 protein levels (19). Disordered segments lack motifs and act directly to regulate protein half-lives by forming initiation sites for degradation by the proteasome (34, 48, 49, 50). Intrinsic disorders can bring about robust proteolytic degradation by (1) easily adopting an extended conformation required by the protease active site; (2) accessing the narrow 20S proteasome gate, which opens and closes in a stochastic manner; and (3) acting as initiator regions for 26S proteasome-dependent degradation, by interacting with the ATPase loops in the 19S subunit and triggering unfolding (37). The deep-seated catalytic residues of the proteasome core particle are only accessible through a long narrow channel, and hence, substrate entry into the proteasome is an important regulatory step of protein half-life (40, 41). A terminal disordered segment of 30 residues or an internal disordered segment of at least 40 residues can span twice this distance and, thus, could be cleaved by the core particle (34). Thus, proteins such as SETD2 that have 17 long disordered segments are expected to be processed quickly owing to the efficient initiation of degradation. Also, the disordered segments are spread across the length of the protein and co-operatively lead to robust degradation of SETD2.

### Variations in the N-terminal region might fine-tune the SETD2 half-life

The gain or loss of disordered segments may be an important contributor to the degradation rate of proteins during evolution (34). Variation in IDRs might be a mechanism for the divergence of half-life among orthologous proteins. IDRs are not required to attain a specified three-dimensional conformation to exert their functional effect and, hence, can undergo mutations without greatly affecting their functionality. Such variation in disordered segments may provide an evolutionary mechanism for fine-tuning protein turnover rates. These forces might lead to inter- as well as intraspecies divergence of protein half-life. Analysis of *Drosophila* SETD2 revealed large and abundant disordered regions (Fig. 1*G*). The differences in the degree of disorder between *Drosophila* and human SETD2 proteins might lead to differences in their half-lives much like what we observed between SETD2 and ySet2. Differences in half-lives between Set2 homologs might be required to adjust for the differences in the deposition mechanism of H3K36 methylation that we speculated previously (19).

In addition to interspecies differences, intraspecies variation in the length of IDRs may arise through mechanisms such as repeat expansion, alternative splicing, and alternative transcription start sites. We indeed found that splice variants and mutations that result in SETD2 mutants with lesser IDRs have higher accumulation as compared with WT SETD2 (Fig. 5). Differences in protein turnover may disturb protein abundance and could lead to diseases (68, 69).

### SETD2 forms *in vivo* liquid droplets

We found that full-length SETD2, in which IDRs are prevalent, forms *in vivo* liquid droplets. IDRs are known to promote phase separation of proteins. Previous studies showed that, when highly expressed, IDRs formed puncta in the nucleoplasm independent of DNA, and in these nucleoplasmic puncta IDR–IDR interaction dynamics were even greater (54, 70). Such IDR hubs facilitate further protein recruitment and by promoting dynamic IDR–IDR interactions can concentrate transcription regulators to establish a transcription-competent compartment (54). Furthermore, phase separation can result in the formation of RNA granules that control the stability, localization, and translation of RNA cargo (71).

Although both SETD2 A and SETD2 C consist of IDRs, only SETD2 C, which contains the enzymatic domains, can phase separate (Fig. 4). It is still not clear how the primary sequence of IDR-containing proteins controls their phase separation. A study that investigated the properties of 22 IDR-containing proteins of the FUS family revealed that only three of these proteins were capable of phase separation (72). Inter-IDR Tyr–Tyr interactions could also promote phase separation but only at high protein concentrations (72). Noticeably, however, SETD2 A (3.2%) and SETD2 C (3.3%) contain a very similar fraction of tyrosine residues. In fact, the SETD2 A region has a greater proportion of residues that are predicted to be disordered than SETD2 C. It is possible that, without the functional domains of SETD2, the IDR-only truncations are not capable of phase separating and are aggregated into a solid state. Another possibility is that these truncations did once phase separate and were liquids but then aged into nondynamic solid aggregates over the course of expressing these truncations. In this case the phase-separated state would be very short-lived and would be impossible to detect. More studies are needed to dissect the role of discrete protein regions and amino acids in regulating IDR-containing proteins’ behavior.

Previously, we and others have shown that SETD2 directly interacts with RNA Pol II and the mRNA processing hnRNPs to regulate gene expression and alternative splicing (9, 21, 51). We found that, even upon deletion of the domains responsible for interaction with Pol II and hnRNPs, SETD2 continues to phase separate and show liquid-like behavior *via* FRAP, but the viscosity of these puncta was much higher than the FL protein and, thus, the FRAP recovery is quite slow. In addition, the puncta for the SRI and SHI deletion mutants showed nuclear puncta that have an internal structure (holes in fluorescence). This heterogeneity is maintained in their positions over time even while the puncta’s fluorescence recovers over time. This suggests that these aggregates may have a solid-like internal core that is filled with mobile protein, much like a hydrogel. It is likely that a combination of intrinsic structural features as well the interactome of SETD2 governs its phase separation.

**Figure 6:**
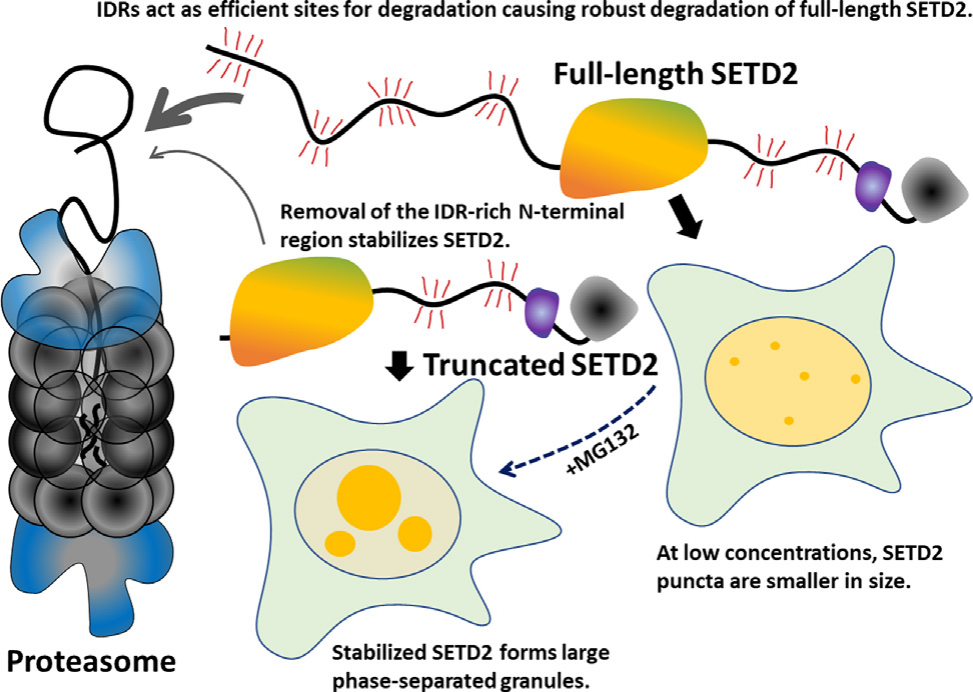
**Cartoon summarizing the findings of this article.** The thickness of the *gray arrow* shows the ease of access to the proteasome. The *red whiskers* depict the sensitivity to proteolysis.

To summarize, SETD2 has multiple long IDRs spread across its length (Fig. 6). These IDRs make it protease sensitive and facilitate its robust proteasomal degradation. Removal of the IDR-rich N-terminal region of SETD2 reduces its protease sensitivity resulting in higher accumulation. Higher accumulation also is associated with its tendency to form larger phase-separated liquid droplets and also results in activator-independent histone methylation. As with super-enhancers (70), SETD2’s phase separation property might facilitate its role in regulating transcription and splicing, which will be interesting to investigate in future studies.

## Experimental procedures

### Plasmids

SETD2-HaloTag human ORF in pFN21A was procured from Promega. Deletion mutants of SETD2 were constructed by PCR (Phusion polymerase, NEB) using full-length SETD2 as a template, and individual fragments were cloned. All constructs generated were confirmed by sequencing. pCDNA3-ySet2 was procured from Addgene.

### Cell line maintenance and drug treatment

HEK293T cells were procured from American Type Culture Collection. Cells were maintained in Dulbecco's modified Eagle's medium supplemented with 10% fetal bovine serum and 2 mM L-glutamine at 37 °C with 5% CO_2_. MG132 (Sigma) was added at a final concentration of 10 μM for 12 h. Cycloheximide (Sigma) was added at a final concentration of 10 μM. Transfections were performed at cell confluency of 40% using Fugene HD (Promega) using a ratio of 1:4 of the plasmid (μg) to transfection reagent (μl).

### Mass spectrometry analysis

N-terminally Halo-tagged deletions of human SETD2 consisted of amino acids 1 to 1692, 1 to 503, 504 to 1403, 1404 to 2564, 1404 to 1963, and 1964 to 2564. Deletion mutants were expressed in HEK293T cells and affinity purified, each in biological duplicates, along with two negative controls. Eluted proteins were trichloroacetic acid precipitated and were analyzed independently by Multidimensional Protein Identification Technology (MudPIT) (73, 74). Briefly, precipitated protein samples were resuspended in 100 mM Tris pH 8.5, 8 M urea to denature the proteins. Proteins were reduced and alkylated before digestion with recombinant LysC (Promega) and trypsin (Promega). Reactions were quenched by the addition of formic acid to a final concentration of 5%. Peptide samples were pressure loaded onto 100 μm fused silica microcapillary columns packed first with 9 cm of 5-μm Aqua C18 reverse-phase material (Phenomenex), followed by 3 cm of 5-μm Luna strong cation exchange material (Phenomenex), followed by 1 cm of Aqua C18 RP. The loaded microcapillary columns were placed in-line with 1200 or 1260 quaternary HPLCs (Agilent). The application of a 2.5-kV distal voltage electrosprayed the eluting peptides directly into LTQ linear ion trap or Velos-Orbitraps mass spectrometers (Thermo Scientific) equipped with a custom-made nano-LC electrospray ionization source. Full MS spectra were recorded on the eluting peptides over a 400- to 1600-*m/z* range, followed by fragmentation in the ion trap (at 35% collision energy) on the first to fifth most intense ions selected from the full MS spectrum. Dynamic exclusion was enabled for 120 s (75). Mass spectrometer scan functions and HPLC solvent gradients were controlled by the XCalibur data system (Thermo Scientific).

RAW files were extracted into .ms2 file format (76) using RawDistiller v. 1.0, in-house developed software (77). MS/MS spectra were searched using ProLuCID (78) with a 500-ppm mass tolerance for peptide and fragment ions. Trypsin specificity was imposed on both ends of candidate peptides during the search against a protein database combining 44,080 human proteins (NCBI 2019-11-03 release), the amino acid sequences for the SETD2 deletion mutants, as well as 426 common contaminants such as human keratins, IgGs, and proteolytic enzymes. To estimate false discovery rates (FDRs), each protein sequence was randomized (keeping the same amino acid composition and length) and the resulting “shuffled” sequences were added to the database, for a total search space of 89,038 amino acid sequences. A mass of 57.0125 Da was added as a static modification to cysteine residues and 15.9949 Da was differentially added to methionine residues.

DTASelect v.1.9 (79) was used in combination with an in-house software, swallow v1.0 (https://github.com/tzw-wen/kite), to select and sort peptide/spectrum matches to FDRs of less than 1% at the spectral, peptide, and protein levels. Spectral-, peptide-, and protein-level FDRs were, on average, 0.18 ± 0.14%, 0.16 ± 0.11%, and 0.53 ± 0.28%, respectively. Results from each sample were merged and compared using CONTRAST (79). Combining all replicates, proteins had to be detected by at least two peptides. Proteins that were subsets of others were removed using the parsimony option in DTASelect on the proteins detected after merging all runs. Proteins that were identified by the same set of peptides (including at least one peptide unique to such protein group to distinguish between isoforms) were grouped together, and one accession number was arbitrarily considered as representative of each protein group.

NSAF7 (80) was used to create the final reports on all detected peptides and nonredundant proteins identified across the different runs. SETD2 deletion mutants, which are subsets of the full-length protein, were added to their respective affinity purifications and their dNSAF values were calculated taking into account their shorter lengths. QPROT (81) was used to calculate log-fold changes, Z-scores and FDR adjusted *p*-values for the SETD2 deletion mutant samples compared with the mock controls.

### FRAP assay

Cells expressing the C4 and N3 GFP tagged fragments of SETD2 were plated into coverslip bottom Mat-Tek dishes. The cells were then imaged using a CSU-W1 spinning disc Ti2 microscope (Nikon) through a 100x Plan Apochromat objective (NA 1.45). Excitation of GFP occurred at 488 nm, and the emission was collected for 50 to 200 ms per frame through a standard FitC filter onto a Flash 4 camera (Hamamatsu). Photobleaching was achieved using a diffraction-limited 488-nm laser beam focused on the region of interest and scanned across that ROI until fluorescence was eliminated. The settings for bleaching were adjusted so that the bleaching step was nearly instantaneous (<2 s). Prebleach images were acquired for ∼0.7 s, and the recovery after bleaching was recorded every 50 to 200 ms for 10 s. Recovery curves were then generated using Fiji (https://imagej.net/Fiji). First, the images were cropped to the cell of interest and then registered to remove the cell/punctum movement using a plugin called Stackregj. After this, an ROI was placed over the bleached portion of the cell and the mean intensity of the ROI was plotted using a plugin called “create spectrum jru v1.” Once all the curves for a particular condition were collected, the curves were all combined into one window using “combine all trajectories jru v1.” The curves were then all normalized to the min and max of the curve using “normalize trajectories jru v1.” The curves were then manually aligned in time so that the bleach point all aligned at the same timepoint. Finally, each curve was fit individually using “batch FRAP fit jru v1.” The fit parameters were then averaged to give the tau and percent recovery.

### Isolation of total RNA and PCR

Total RNA was extracted from cells as per the manufacturer’s (Qiagen) instructions. It was further treated with DNaseI (NEB) for 30 min at 72 °C to degrade any possible DNA contamination. RNA (2 μg) was subjected to reverse transcription using the QScript cDNA synthesis mix according to the manufacturer’s instructions. cDNAs were then amplified with the corresponding gene-specific primer sets. For RT-PCR, PCR was conducted for 24 cycles using the condition of 30 s at 94 °C, 30 s at 60 °C, and 30 s at 72 °C. The PCR products were analyzed on 1% agarose gels containing 0.5 μg/ml ethidium bromide. The sequence of oligos is in Fig. S6.

### Antibodies

Halo (Promega, G9211), β-actin (Abcam, ab8224), SETD2 (Abclonal, A3194), FLAG (Sigma-Aldrich, A8592) were the antibodies used.

## Data availability

All relevant data are available and are contained within the article. The complete mass spectrometry datasets for SETD2 fragments, as well as mock affinity purification negative controls, have been deposited to the Proteome Xchange (PXD026571) and may be accessed through the MassIVE repository *via* ftp://massive.ucsd.edu/ (MSV000087587). Original data underlying this manuscript can be accessed from the Stowers Original Data Repository at http://www.stowers.org/research/publications/libpb-1648.

## Supporting information

This article contains supporting information.

## Conflict of interest

The authors declare that they have no conflicts of interest with the contents of this article.
