# Supplementary material for: The disordered regions of the methyltransferase SETD2 govern its function by regulating its proteolysis and phase separation
Source: J Biol Chem. 2021 Aug 13;297(3):101075. doi: 10.1016/j.jbc.2021.101075 (PMC8405934; doi:10.1016/j.jbc.2021.101075)
Supplement: Supplemental Figures S1–S6 [file mmc2.pdf]

The disordered regions of the methyltransferase SETD2 govern its function by regulating its proteolysis and phase separation

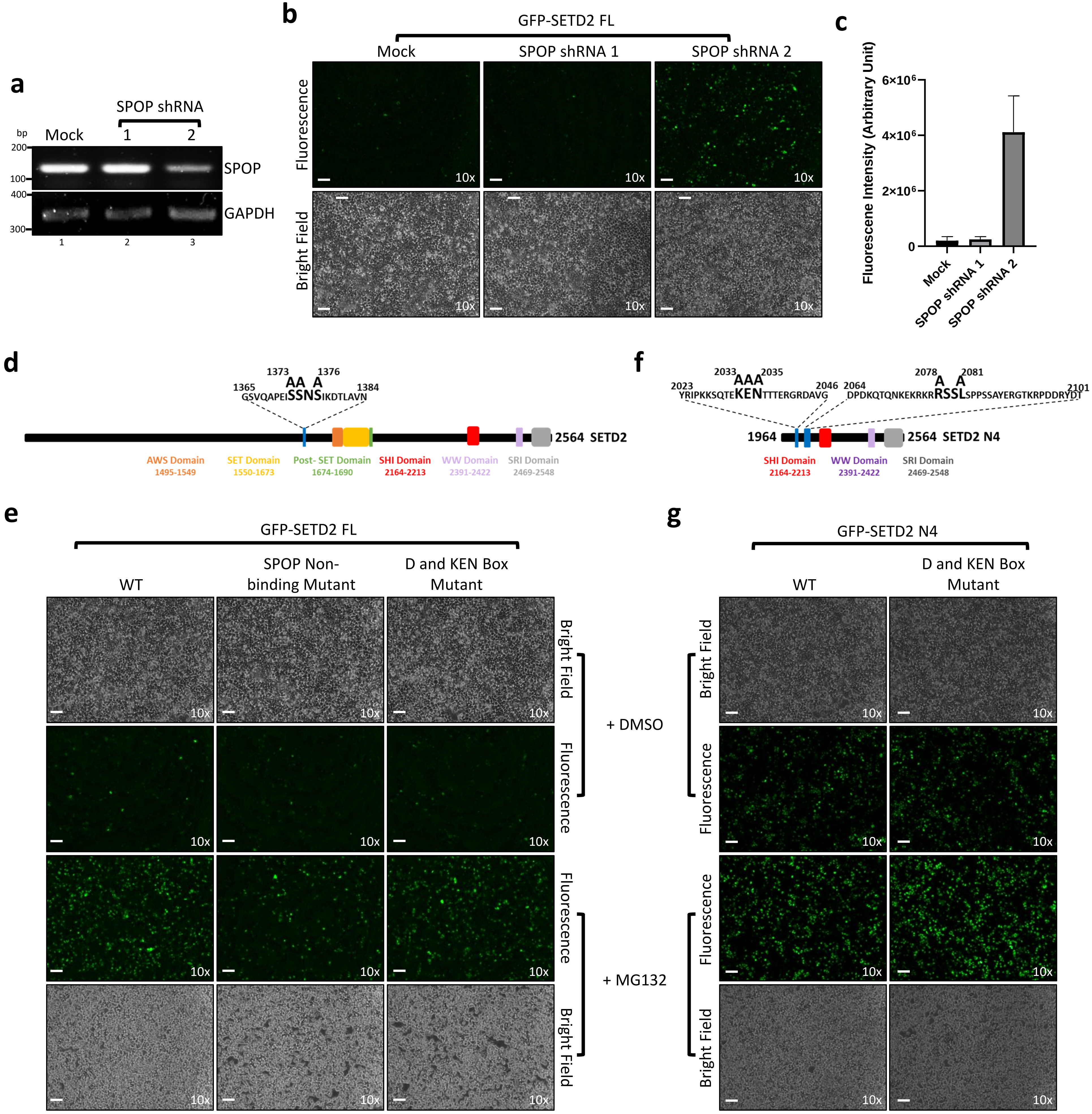

a
SPOP shRNA
bp
Mock 1 2
200
100
400
300
SPOP
GAPDH
1 2 3
b
GFP-SETD2 FL
Mock
SPOP shRNA 1
SPOP shRNA 2
Fluorescence
Bright Field
10x
c
Fluorescene Intensity (Arbitrary Unit)
6×10⁶
4×10⁶
2×10⁶
0
Mock
SPOP shRNA 1
SPOP shRNA 2
d
1373 1376
1365 AA A 1384
GSVQAPEISSNSIKDTLAVN
2564 SETD2
AWS Domain 1495-1549
SET Domain 1550-1673
Post- SET Domain 1674-1690
SHI Domain 2164-2213
WW Domain 2391-2422
SRI Domain 2469-2548
f
2033 2035 2078 2081
2023 AAA 2046 2064 A A 2101
YRIPKKSQTEKENTTTERGRDAVG DPDKQTQNKEKRKRRSSLSPPSSAYERGTKRPDDRYDT
1964
2564 SETD2 N4
SHI Domain 2164-2213
WW Domain 2391-2422
SRI Domain 2469-2548
e
GFP-SETD2 FL
WT
SPOP Non-binding Mutant
D and KEN Box Mutant
Bright Field
Fluorescence
+ DMSO
+ MG132
10x
g
GFP-SETD2 N4
WT
D and KEN Box Mutant

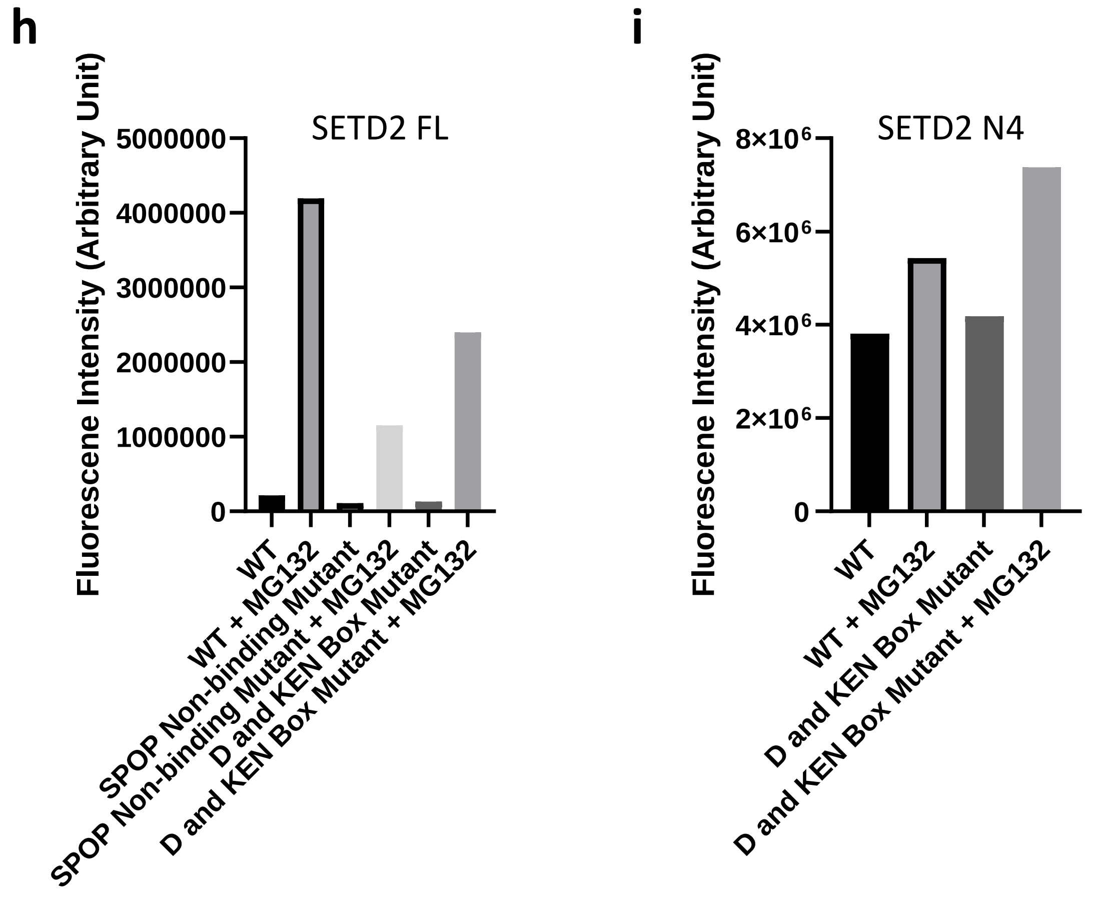

Figure S1: (a) RNA was isolated from GFP-SETD2 FL expressing 293T cells transfected with SPOP shRNA and RT-PCR was performed to check transcript levels. GAPDH was used as a normalization control. (b, c) Microscopy images and their quantification showing the effect of SPOP shRNA treatment on expression of GFP-SETD2 FL in 293T cells. The scale bar in is 1 mm. (d, f) Cartoon depicting the position and sequence of SPOP binding and putative D/KEN Box in SETD2 and the mutation introduced to disrupt those. (e, g, h, i) Microscopy images and their quantification showing expression of GFP-SETD2 constructs in 293T cells and the effect of MG132 treatment on their expression. The scale bar in is 1 mm. WT- Wild Type. Please note that the microscopy settings used to capture images in panels b, e and g were different to get clear pictures while avoiding saturation and thus, cannot be compared amongst themselves.

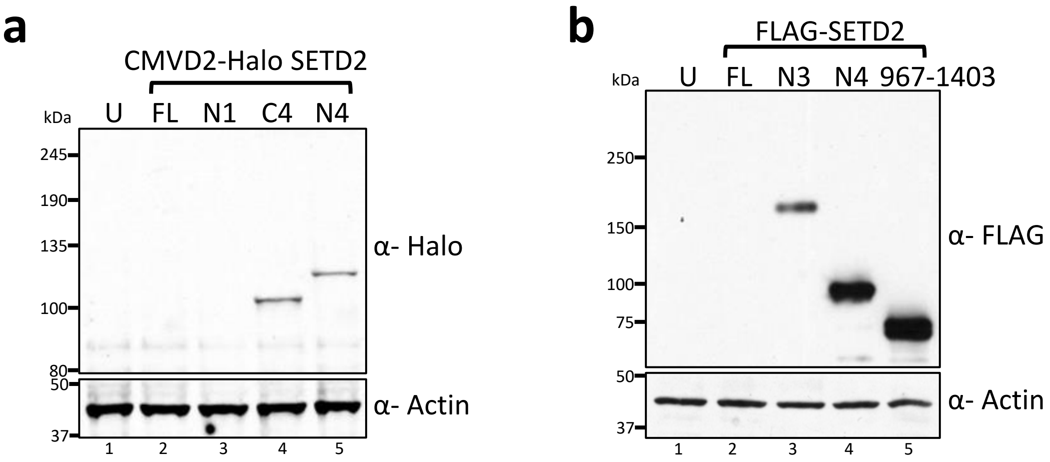

Figure S2: Western blot of whole-cell lysates of 293T cells expressing (a) Halo-tagged SETD2 constructs under CMVD2 promoter and (b) FLAG-tagged SETD2 constructs under CMV promoter.

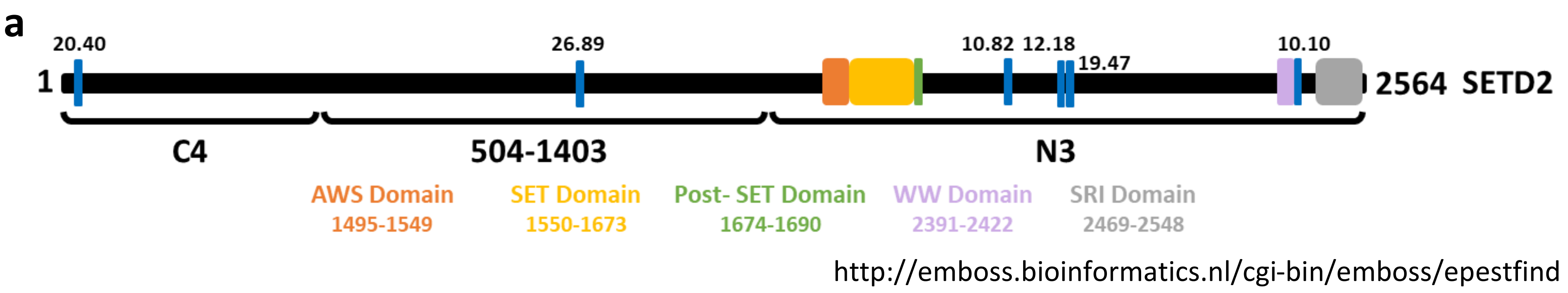

b

Position	Sequence	Score
20-31	HPTPEEEENEAK	20.40
96-115	KQSDTPNPPAVPLQVDSTPK	5.17
117-134	KMEIGDTLSTAEESSPPK	8.87
169-216	HAAPLPAVIAESTTVDSPPSSPPPPPPPAQ ATTLSSPAPVTEPVALPH	9.62
1035-1063	HEDYSGSSESSNDESDSEDTDSDDSSIPR	26.89
1074-1089	KNSTLPMEETSPCSSR	6.78
1831-1850	KTAVPPLSEGDGYSSENTSR	5.5
1852-1863	HTPLNTPDPSTK	8.28
1863-1874	KLSTEADTDTPK	10.82
1948-1963	KLPTSEPEADAEIEPK	12.18
1969-1994	KLEEPINEETPSQDEEEGVSDVESER	19.47
2413-2433	RQTQWDPPTWESPGDDASLEH	10.12

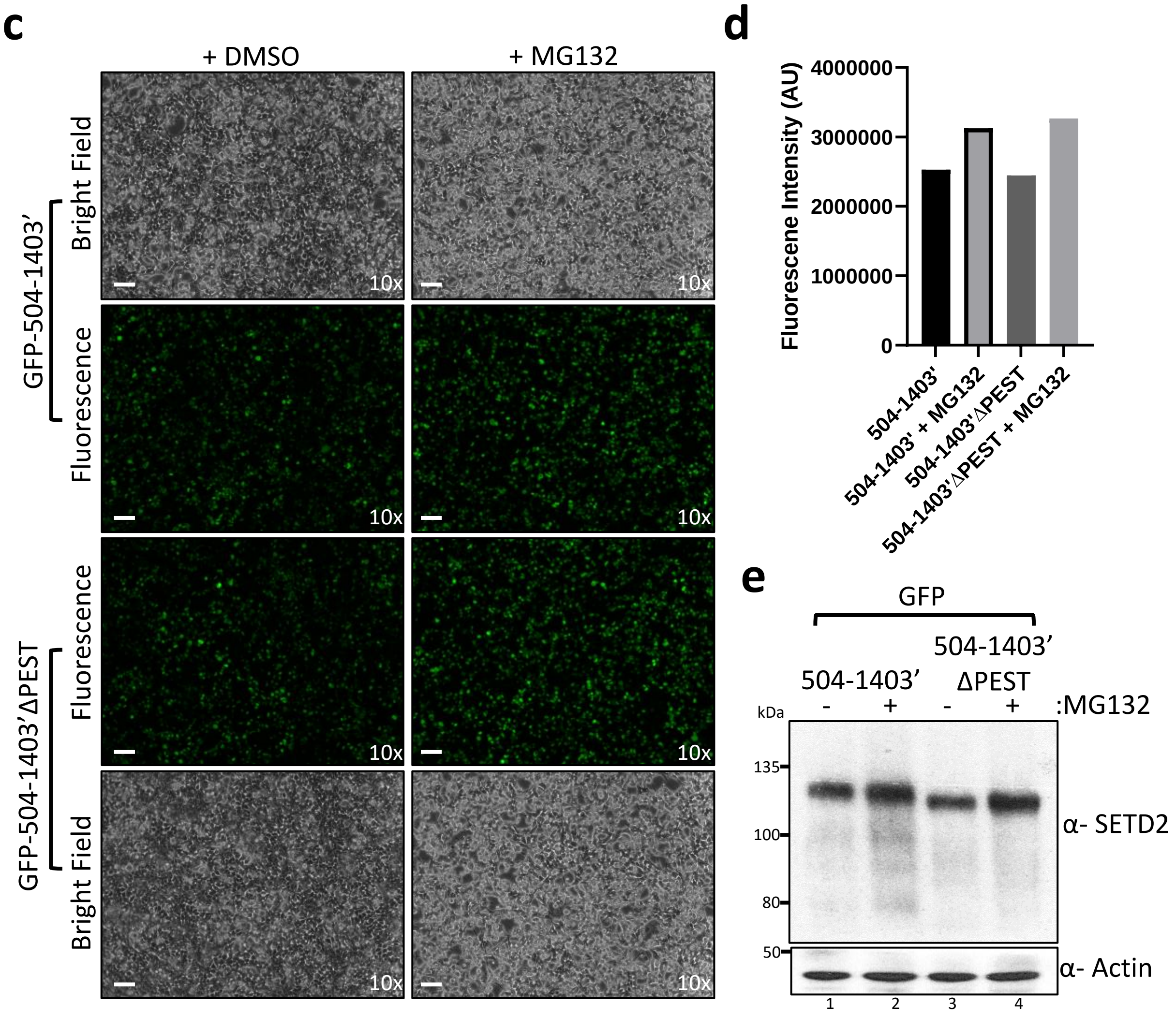

Figure S3: (a) Cartoon illustrating the location of PEST motifs in SETD2 of score >10 (>5 is considered significant). (b) Position, sequence and score of PEST motifs in SETD2. (c, d) Microscopy images and their quantification showing expression of GFP-SETD2 fragments. See text for more details. The scale bar in is 1 mm. (e) Western blot of whole-cell lysates of cells shown in (c) probed with the antibodies indicated.

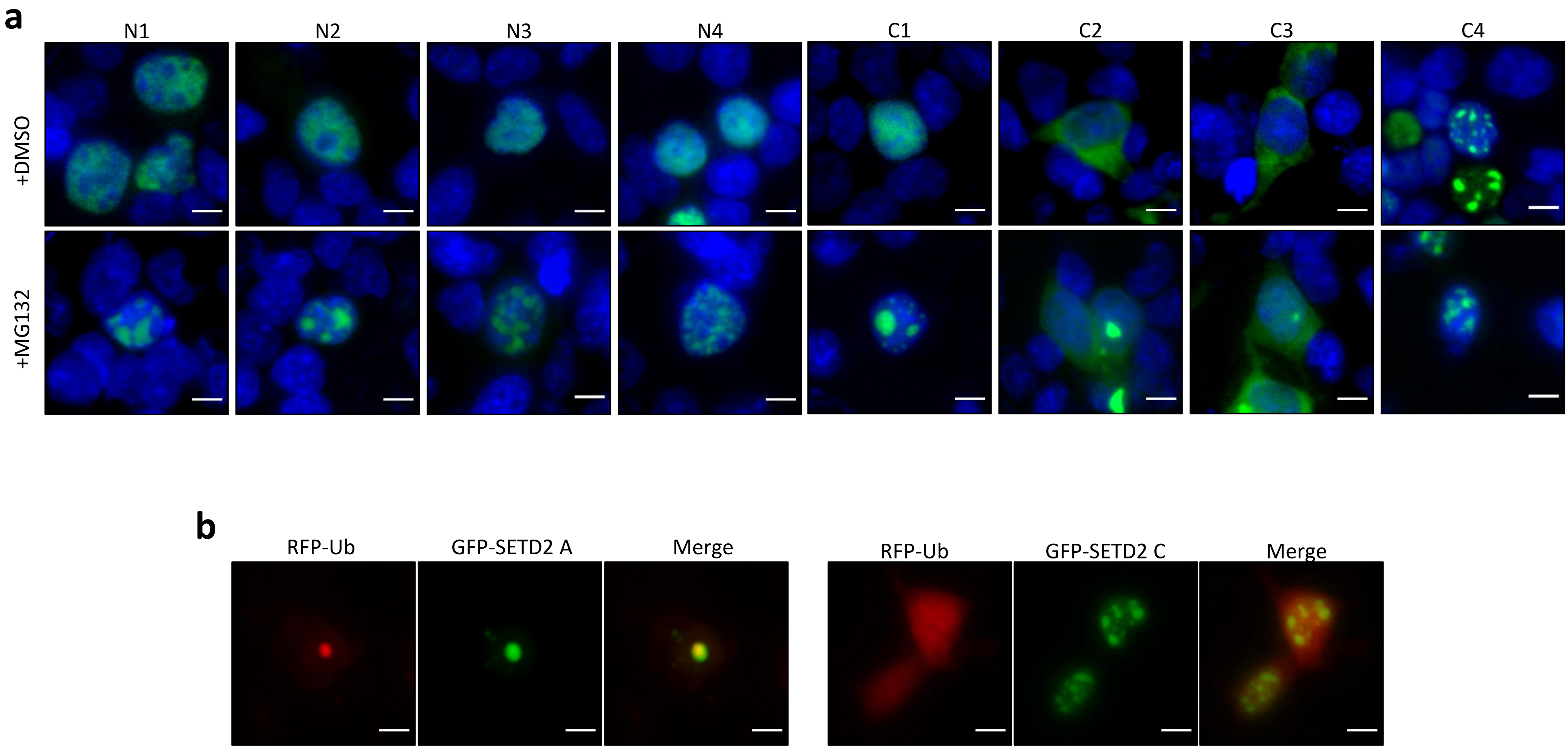

Figure S4: (a) Microscopy images showing that GFP-SETD2 truncations form puncta especially upon MG132 treatment. (b) Microscopy images showing the localization of RFP-Ub with GFP-SETD2 A and C. The scale bar is 10 μm.

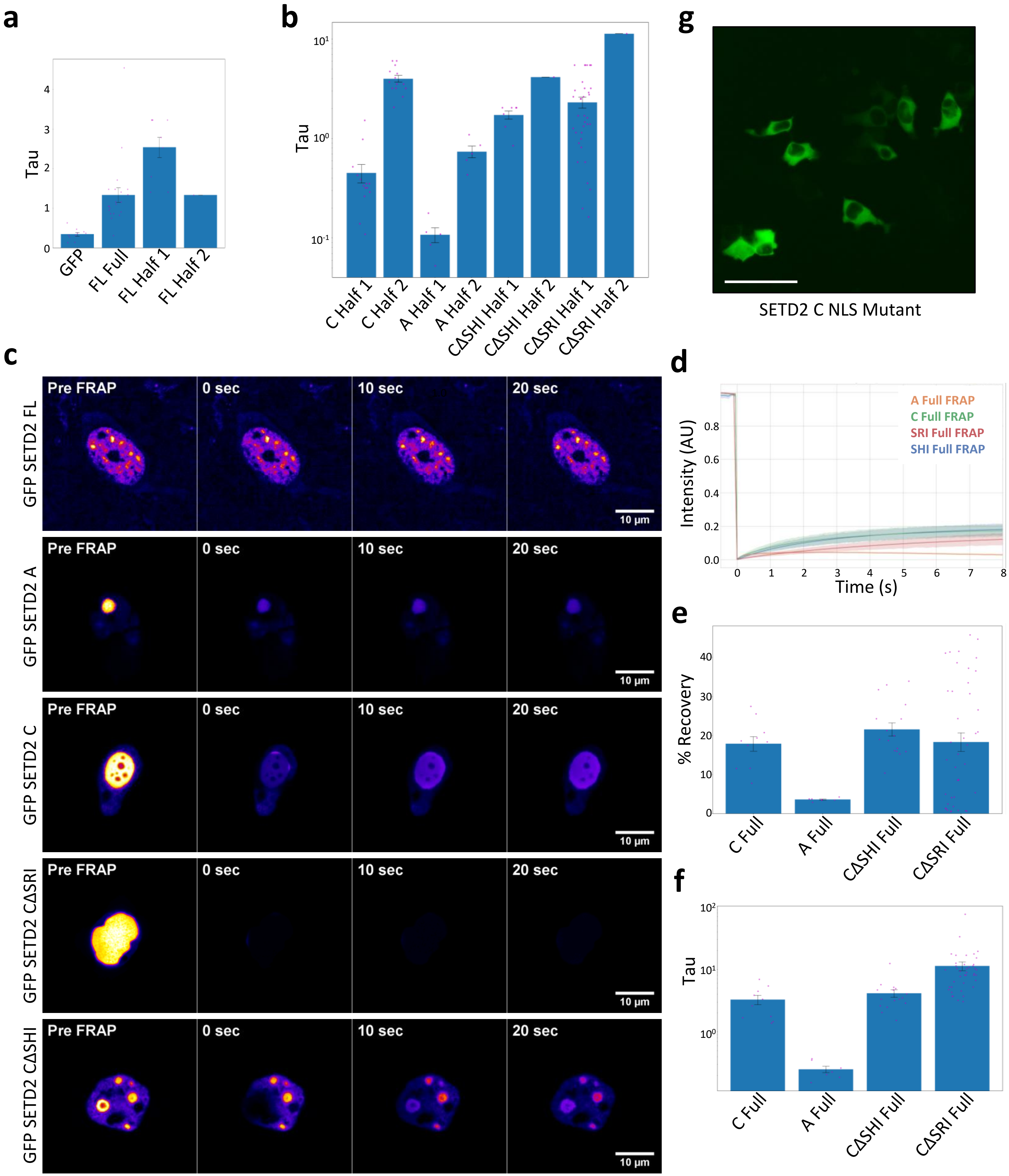

Figure S5: (a, b, d-f) Plots and bar graphs showing the recovery of fluorescence in the bleached region of interest and the Tau values calculated from the FRAP experiments. (c) Microscopy images showing different time points of the FRAP experiments. The scale bar is 10 µm. (g) Microscopy image of NLS mutant of SETD2 C. The scale bar is 100 µm.

Oligo	Sequence (5’-3’)
SPOP_F	TACCCTCTTCTGCGAGGTGA
SPOP_R	CGGGAATTCTCCCACAGTCC
GAPDH _F	TTCGACAGTCAGCCGCATCTTCTT
GAPDH _R	CAGGCGCCCAATACGACCAAATC
SPOP_shRNA_1 F	CCGGGATTCAAGAAATTCATCCGTAGATATCTACGGATGAATTTCTTGAATCTTTTTG
SPOP_shRNA_1 R	AATTCAAAAAGATTCAAGAAATTCATCCGTAGATATCTACGGATGAATTTCTTGAATC
SPOP_shRNA_2 F	CCGGTTCCAGGCTCACAAGGCTATCGATATCGATAGCCTTGTGAGCCTGGAATTTTTG
SPOP_shRNA_2 R	AATTCAAAAATTCCAGGCTCACAAGGCTATCGATATCGATAGCCTTGTGAGCCTGGAA

Figure S6: Sequence of oligos used to perform RT-PCR and knockdown.
